# Fabrication and Characterization of Polyvinylpyrrolidone-Eggshell Membrane-Reduced Graphene Oxide Nanofibers for Tissue Engineering Applications

**DOI:** 10.3390/polym13060913

**Published:** 2021-03-16

**Authors:** Shahnaz Ghorbanzadeh Sheish, Rahmatollah Emadi, Mehdi Ahmadian, Sorour Sadeghzade, Fariborz Tavangarian

**Affiliations:** 1 Materials Engineering Group, Pardis College, Isfahan University of Technology, Isfahan 84156-83111, Iran; s.ghorbanzadeh@pa.iut.ac.ir; 2 Department of Materials Engineering, Isfahan University of Technology, Isfahan 84156-83111, Iran; remadi@cc.iut.ac.ir (R.E.); ahmadian@cc.iut.ac.ir (M.A.); 3 Mechanical Engineering Program, School of Science, Engineering and Technology, Penn State Harrisburg, Middletown, PA 17057, USA

**Keywords:** electrospinning, polyvinylpyrrolidone, eggshell membrane, graphene oxide, biocompatibility, wound dressing

## Abstract

One of the best methods to prevent wound infection and speed up wound healing is wound dressing based on nanofiber–polymer scaffolds, which have acceptable antimicrobial performance and appropriate skin regeneration capabilities. In this paper, the electrospinning method was applied to synthesize the polyvinylpyrrolidone-acrylic acid hydrogel (PVPA)–eggshell membrane (ESM)–reduced graphene oxide (rGO) nanosheets nanocomposite dressings with different reduced graphene oxide contents (0, 0.5, 1, and 2 wt.%). Thus, smooth nanofibers were fabricated, including a high amount of rGO, which reduced the fiber diameter. Based on the results, rGO played an important role in water impermeability. The results showed that by increasing the rGO concentration from 0.5 to 2 wt%, the contact angle value increased persistently. Results showed that compared to PVPA–ESM, the mechanical strength and strain of PVPA–ESM/1 wt% rGO significantly enhanced 28% and 23%, respectively. Incorporation of 1 wt% rGO enhanced swelling ratio from 875% for PVPA-ESM to 1235% after 420 min, while increasing the rGO to 2 wt% increased the degradation rate of the composites. According to the in vitro cell culture studies, PVPA-ESM wound dressings with 0.5–1 wt% rGO content enhanced PC12 cell viability compared to the wound dressings without rGO nanosheets. Generally, rGO–loaded PVPA-ESM nanofiber wound dressing can be considered as a potential candidate to be used in skin regeneration applications.

## 1. Introduction

The first protective obstacle to the external environment is human skin, which defends the body against microbial intrusion and ample water loss [1,2,3]. Patients suffering from burn injuries and those who have had surgeries are vulnerable to systemic immunosuppression and are at a high risk of infection [4,5]. Approximately two-thirds of deaths in these patients are due to the aforementioned conditions [6,7,8]. Moreover, one of the greatest threats to human health is the infection issuance due to drug-resistant pathogens [9,10]. Consequently, researchers are more persuaded to suggest different remedies [11]. One of the low-cost methods to generate fibrous polymers for wound dressing is electro-spinning. By selecting the correct materials in this method, some biological substitutes can be produced for skin defects. By controlling the size and percentages of porosities and creating substantial surface-area-to-volume ratios, this method has prominent features beneficial for the biomedical fields [12,13,14].

Polyvinylpyrrolidone (PVP) has been introduced as a synthetic polymer with high biocompatibility, chemical stability, great film-forming ability, and mechanical properties [15,16]. PVP-based hydrogels have an important role in wound dressing application [17]. These macromolecular systems can incorporate an aqueous medium. Wetting capabilities, architecture, and their structure properties can influence the faster healing of skin tissue regeneration [18]. The softness and high-water content of PVP hydrogels resembles natural living tissues more than any other type of synthetic biomaterial, which also contributes to their biodegradability and biocompatibility [19]. However, PVP hydrogels are limited in their usage due to their weak mechanical properties. To increase its mechanical properties, PVP, and its monomer, *N*–vinyl pyrrolidone have been copolymerized with acrylic acid and other vinyl monomers [20,21]. Incorporating the reinforcement agents into the polymer matrix prevents the diffusion of substances through the membrane [22,23]. The performance of these multicomponent systems can be optimized by their synergic features [24]. On the other hand, GO is more appropriate for biomedical applications because of the hydrophilicity that is provided by hydroxyl and carboxyl groups [25,26]. These groups enable GO [27] to be functionalized by a range of synthetic and natural molecules, including proteins, polymers, nanoparticles, and small molecules [28]. The cytotoxicity of GO-functionalized materials can be reduced by protein [29]. To acquire hybrid composites with great material features and electrical conductivity, researchers in previous studies have used polymers with graphene-based nanomaterials, with excellent mechanical and physical properties [30]. Many polymer-GO composite nanofibers have been produced in recent years [31]. Pant et al. [32] made nylon-6 spider-wavelike nano-nets via embedding proper levels of GO in the polymer solution by using electrospinning [33,34]. As a result, the hydrogen bond formation between polymer molecules and the GO sheet leads to the nice dispersing of the GO nanosheet in the nylon-6 solution. PVP–GO nanofibers were fabricated via electrospinning by Liu [35]. Although the electrospinning of synthetic PVP-based nanofiber is complex, it needs a sizeable organic solvency content [36]. Due to the high consumption of hen eggs around the world, a large amount of discarded egg wastes consists of eggshell (ES) and eggshell membrane (ESM) is available [37]. ES and ESM are inorganic and organic materials that have a wide range of applications such as soil conditioner, initial material for the synthesis of bioceramics and collagen, and recently for wound dressing [38]. Among these applications, much interest has been devoted to the use of eggshell powder as reinforcement in polymer industries. Saeb et al. [39] utilized hen eggshell due to the peptide functional groups and proteins in its structure to create a material for curing aid purposes when added to the epoxy resin. In another study, Subramani Bhagavatheswaran et al. [40] mentioned that adding ES (with superior properties and low price) biowaste powder in polymer matrix can develop a biodegradable polymer which can reduce the contaminants in environment. They fabricated a new biodegradable acrylonitrile butadiene rubber (NBR) reinforced with ES and CaCO_3_ micro-fillers with improved mechanical properties [40]. Eggshell membrane (ESM) is an inorganic waste material containing more than 500 proteins and peptides, including collagens, glycoproteins, etc. [41]. ESM attract much attention in recent years due to its biological functions such as anti-adhesive, antioxidant properties, anticancer, and antimicrobial [42]. Furthermore, ESM has appropriate properties for medical applications such as suitable moisture retention, air permeability, cell response action of amino acids, and the ability to attach to textured surfaces due to its network structure [43,44]. Researchers [43] have used ESM as a biodegradable bone reproduction inhibitor, a media for biosorption, and a template for forming ordered tube networks [43]. Moreover, recently, ESM powder was used as wound dressing in a mouse excisional which accelerated the wound closure, improved tissue formation, and accelerated the deposition of collagen at the wound location [42]. Blending biopolymers with synthetic polymers to produce new polymeric materials with better properties received great attention [44]. To design the appropriate wound dressing with high biological and mechanical properties, a mixture of ESM, PVP, and GO nanosheets can be a good idea due to the aforementioned properties. The aim of this paper was to develop wound dressings composed of PVPA–ESM/rGO nanosheets. PVPA–ESM/rGO nanofibers were fabricated via electrospinning technique. Then, they were tested in vitro to determine their capability in the wound healing process. ESM was selected as the base material for electrospinning in aqueous media. PVP’s electrospinning processability was chosen as the supporting polymer for incorporating the ESM. The effects of adding rGO nanosheets into the PVPA-ESM nanofibers composite on the biological, mechanical, and wettability of these nanofibers composite was evaluated.

## 2. Materials and Methods

### 2.1. Materials

Polyvinylpyrrolidone (PVP, (C_6_H_9_NO)_n_, M_w_ = 40,000 g/mol) was purchased from Sigma-Aldrich Co., Cleveland, OH, USA. Acrylic acid (CH_2_=CHCOOH, M_w_ = 72/06 g/mol), and methylene bisacrylamide (C_7_H_10_N_2_O_2_, M_w_ = 154.17 g/mol), and potassium persulfate (K_2_S_2_O_8_, M_w_ = 270.322 g/mol) were used as monomer, cross-linking agent, and initiator, respectively, and were purchased from Sigma–Aldrich Co., Cleveland, OH, USA. Triton^™^ X-100 was purchased from Sigma-Aldrich Co., Cleveland, OH, USA (C_14_H_22_O(C_2_H_4_O)_n_ (n = 9–10), M_w_ = 647 g mol^−1^) as surfactant. Graphite powder, H_2_SO_4_, H_3_PO_4_, KMnO_4_, H_2_O_2_ (30%), and hydrazine hydrate were purchased from Sigma-Aldrich Co., Cleveland, OH, USA. Glycerol and CaCl_2_ were attained from Merck Co., Munich Germany. Hen eggs were bought from the local market (Isfahan, Iran). Deionized (DI) was used in all parts of the experiment.

### 2.2. Preparation of the Eggshell Membrane (ESM)

First, the egg white and yolk were removed through a small piercing at the blunt tip of the egg. Then, after washing the eggshell with deionized water and to separate ESM from the shell, it was soaked in HCl (1 M) solution at 25 °C for 1 h. In the next step, the ESM was rinsed using deionized water. The extracted ESM was attained after drying in a vacuum oven at 40 °C for 24 h. Finally, the membranes were cut into small pieces for preparing the electrospinning solution.

### 2.3. Fabrication and Crosslinking Procedure of PVP/Acrylic Acid-Based Hydrogel

**Figure 1:**
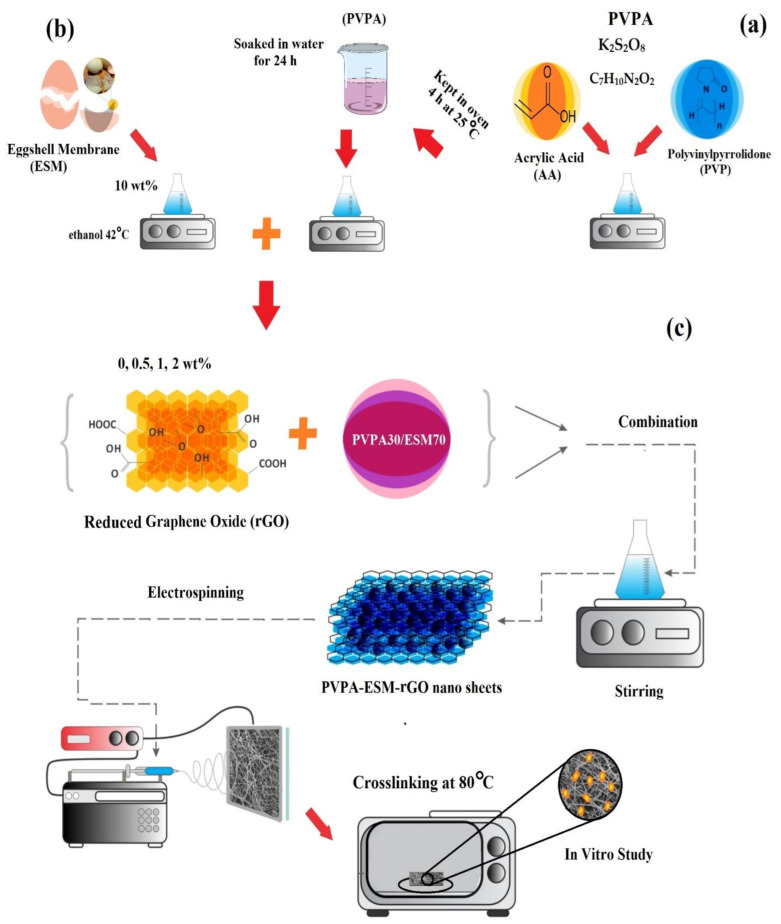
Schematic representation of the fabrication of (**a**) PVP/acrylic acid–based hydrogel, (**b**) eggshell membrane solution, and (**c**) the electrospun PVPA–ESM fibers modified by rGO nanosheets.

Free radical polymerization technique was used to fabricate PVP/acrylic acid–based hydrogel (the obtained material was called PVPA). For this purpose, aqueous solutions of acrylic acid (1 wt. %) was prepared and then 5 g PVP was added to this solution to fabricate PVP/acrylic acid hydrogel (marked as solution A). The cross-linking agent (C_7_H_10_N_2_O_2_) and initiator (K_2_S_2_O_8_) with concentration of 3 *w*/*w* were chosen and were added dropwise into solution A and stirred for 1 h. Finally, the prepared hydrogel solution was kept in oven at 25 °C for 4 h. The hydrogel was then removed and placed in distilled water for 24 h to remove unreacted monomers (Figure 1a).

### 2.4. Synthesis of Reduced Graphene Oxide (rGO)

The GO nanosheets were provided by a modified Hummer’s method as described in [45]. Briefly, graphite and KMnO_4_ (ratio as 1:6 g) were blended with a mixture of H_2_SO_4_ and H_3_PO_4_ acids with a ratio of 180:20 mL and stirred at 50 °C for 12 h. Then, the solution was cooled down at ambient temperature in water solution containing 30% H_2_O_2_. In the next step, the suspension was centrifuged, washed, and then was kept in vacuum oven at 60 °C for 24 h. The chemical reduction method was used to reduced graphene oxide. Hydrazine hydrate was used as a reducing agent, and this mixture was heated at 85 °C for 24 h. The produced reduced graphene oxide (rGO) was used as additive in PVPA–ESM composite.

### 2.5. Fabrication Procedure of PVPA–ESM/rGO Nanofibers

The electrospinning technique was chosen to synthesize the PVPA–ESM/rGO nanocomposite with various amounts of rGO nanosheets. The ESM solution (10 wt.% ethanol–based solution, Figure 1b) was then fabricated and added to the PVPA solution with a *v*/*v* ratio of 70/30. Then, 0, 0.5, 1 and 2 wt.% of rGO nanosheets were added to this solution to enhance spinnability. Before starting the electrospinning process, to improve the suspensions’ spinnability, Triton 100 at the concentration of 0.5 wt.% was added to the suspensions. After that, to have a homogenous dispersion of the rGO nanosheets, the prepared solution was sonicated for 30 min (ES375H BENCH, Hilsonic Ultrasonic Cleaners Co., Cashel Road, Wirral, Merseyside, UK) at an ambient temperature. To fabricate the PVPA–ESM/rGO nanofibers, the prepared suspensions were fed into a 1 mL syringe having a 23 G blunted stainless–steel needle and were electrospun (Figure 1c). Throughout the electrospinning process, the voltage, flow rate, and the tip to the collector distance were considered as 18 kV, 0.12 mL/h, and 15 cm, respectively. To prepare the electrospinning PVPA–ESM/rGO nanofibers, a grounded aluminum foil was used as a collector. After electrospinning and before crosslinking, the fabricated nanofibers were dried in a vacuum desiccator at room temperature for 24 h. At the end, the nanofibers were kept at 80 °C overnight and then soaked in methanol for 1 h to crosslink PVPA for further experiments. A distance of 1 cm was considered between the cage bars and the collector’s rotation speed (800 rpm). Random nanofibers were deposited on the aluminum foil slide placed on the collector plate.

### 2.6. Measurements and Characterizations of PVPA–ESM–rGO Nanofibers

The sessile drop method and a contact angle goniometer (KRUSS, DSA25, Hamburg, Germany) were used to measure the wettability of samples at the room temperature (25 °C). The sessile drop was formed by depositing 2 μL of the test liquid using an automatic micro–syringe. To specify the weight loss of the cross–linked nanofibers, three samples from each model with a weight of about 2 mg were immersed in phosphate–buffered saline (PBS) solution at 37 °C with pH = 7.4, for 1, 7, 14, 21 and 28 days. PBS solution was refreshed every three days and after each timepoint ended, the samples were rinsed with PBS, and then dried and weighed. In order to evaluate the swelling rate of the prepared PVPA–ESM–rGO nanofibers wound dressings with different amount of rGO were soaked in PBS (pH 7.4) solution for different times at ambient temperature. This procedure was continued until no more weight increasing was observed in the samples. The degree of swelling of these composite films at equilibrium was calculated based on following Equation [46]:

Q = (W_w_ − W_d_/W_d_) × 100 (1)

where W_w_ and W_d_ are the weights of swollen and dry samples, respectively, and Q is degree of swelling.

Scanning electron microscopy (SEM, JEOL JSM–6380LA, JEOL (Europe) BV, Nieuw–Vennep, Amsterdam, The Netherlands) was used to study the PVPA–ESM/rGO nanofibers morphology. Furthermore, field emission scanning electron microscopy (FE–SEM, Helios Nanolab 660, an acceleration voltage of up to 30 kV) was carried out to investigate the rGO nanosheets surface morphology. To determine the size of the electrospun nanofibers, Image J software was used. The phase components were determined using an X–ray diffractometer (XRD, Siemens D5000) with Cu–Kα radiation (45 kV, 40 mA, 2θ of 5–80°) with a scanning speed of 4°/min. Attenuated total reflectance–Fourier transform infrared spectroscopy (ATR–FTIR, Bruker tensor) was performed over a range of 600 to 3700 cm^−1^ with a resolution of 2 cm^−1^ to verify the functional groups of the prepared nanofibers.

### 2.7. Cell Culture

To investigate the cell compatibility and cytotoxicity of the nanofiber’s samples, cell culture tests were performed by the extraction method using the PC12 cell lines obtained from Pasteur Institute of Iran. The nanofiber samples were equalized for the experiments based on their weight. After sterilizing the nanofibers samples in ethanol for 2 h, and after rinsing samples with PBS solution (pH = 7.4), the samples were exposed to autoclave. At the end, the samples were immersed in 600 µL of culture medium overnight. The PC12 cells with a cell density of 2 × 10^4^ per culture dish were seeded on samples and kept at 37 °C under 5% CO_2_ condition by refreshing the culture medium every three days. To evaluate samples’ cytotoxicity, the colorimetric analysis was used. After 3 and 7 days of incubation, the culture medium was discarded. The samples were then rinsed with a PBS solution and then immersed in 2.5% glutaraldehyde solution and 0.1% osmium tetroxide for 3 h and 40 min, respectively. The optical density was measured using an Elisa plate reader (STAT FAX 2100, Palm, FL, USA) at 545 nm wavelength. Finally, the mean and standard deviation of the cell viability of each sample were reported [23].

### 2.8. Statistical Analysis

The test results were reported as the mean ± standard error (SE), and to show the significant difference among all data, GraphPad Prism software was used to analyze the data with the *p*–value < 0.05 (*). Data were analyzed for statistically significant differences with 2–way ANOVA and the Turkey test.

## 3. Results and Discussion

### 3.1. Eggshell Membrane Characterization

**Figure 2:**
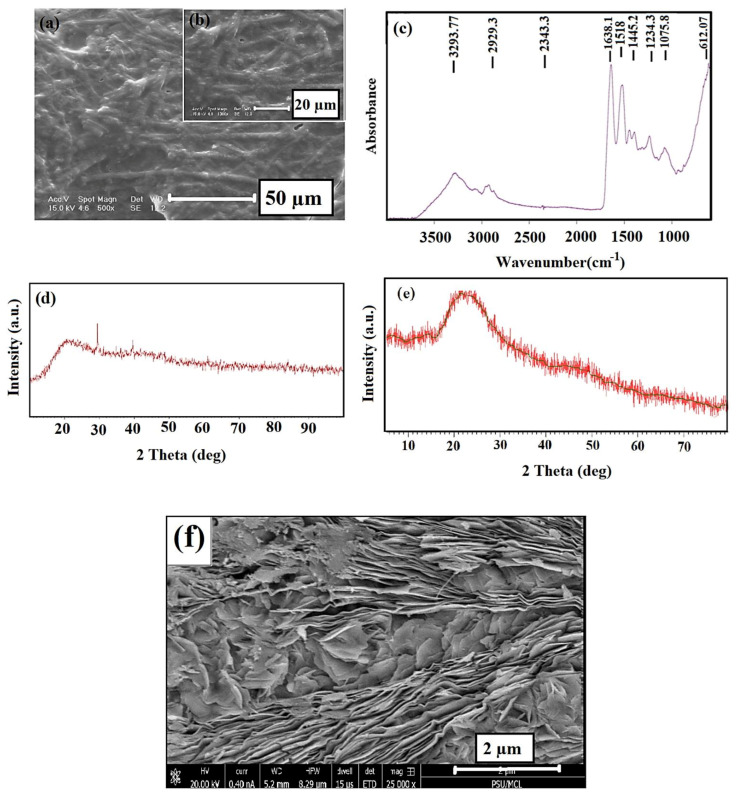
(**a**,**b**) SEM images, (**c**) FTIR spectra, (**d**) XRD pattern of eggshell membrane (ESM), (**e**) XRD pattern, and (**f**) FE–SEM images of rGO.

According to Figure 2a,b which shows the SEM images of ESM, many nanofibers are arranged without forming a specific direction in its network. The FTIR spectra of the prepared ESM specimen (Figure 2c) showed the characteristic peaks of ESM. As seen, the collagen’s functional groups (I, V, X type) can be observed [47,48]. The characteristic peaks were observed at two various areas: the first area is between 3750 and 2500 cm^−1^ and the second area is below 1700 cm^−1^ [49]. The most intense peak in the area with higher wavelengths was observed at 3287 cm^−1^, related to the stretching mode of O–H and N–H groups [36,47]. The peaks at 3060, 2932, and 2869 cm^−1^ are related to the asymmetric stretching vibrations of the C–H and CH_2_ groups, which agree with previous studies. The peaks at 1643 cm^−1^ (C=O stretching), 1536 cm^−1^ (amide N–H bending), 1451 cm^−1^ (CH_2_ scissoring), 1109 cm^−1^ (amine C–N stretching), and 661 cm^−1^ v (C–S) were observed in the lower wavelength region [48]. The amorphous structure of the material is observed by the XRD pattern of the ESM specimen (Figure 2d) in the broad peak at around 20.2° [48] as the composition of the natural ESM specimen includes a lot of amines, amides, and carboxylic compounds, which are mostly amorphous. Figure 2e represents the XRD patterns of rGO nano sheets. The characteristic peak of rGO was observed at 23.2° (002) as reported by Yasin et al. [45]. Furthermore, the FE–SEM image of rGO nanosheets was shown in Figure 2f; as can be observed from Figure 2f, the reduced graphene oxide has a layered structure, with thin and homogeneous graphene plates.

### 3.2. Morphology of the PVP–ESM–rGO Nanofibers

**Figure 3:**
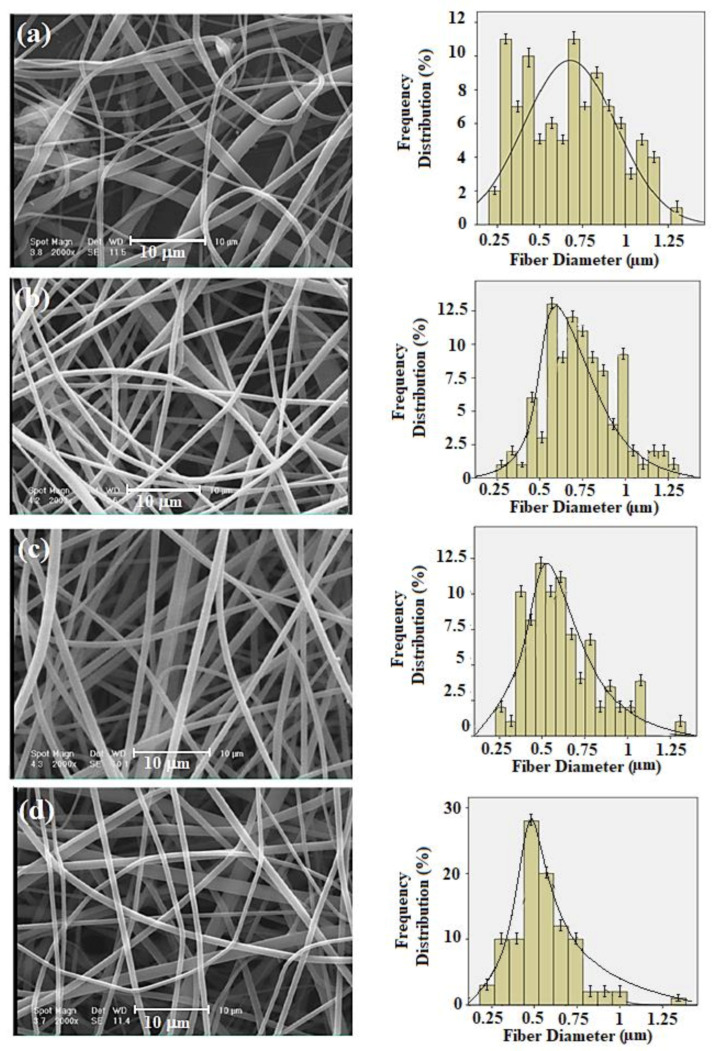
SEM images and frequency distribution of the fiber diameter for (**a**) PVPA–ESM, (**b**) PVPA–ESM/0.5 wt.% rGO, (**c**) PVPA–ESM/1 wt.% rGO, and (**d**) PVPA–ESM/2 wt.% rGO fibers.

The morphologies of the electrospun PVPA–ESM nanofibers with various rGO percentages are shown in SEM images (Figure 3a–d). Smooth and uniform fibers with an average diameter of 687 ± 17 nm were observed in PVPA–ESM (Figure 3a). However, incorporating rGO into PVPA–ESM composite caused the formation of rougher fibers with smaller diameter. In the samples containing 0.5, 1 and 2 wt.% rGO (Figure 3b–d), the fibrous diameters decreased to 552 ± 43 nm, 521 ± 25 nm, and 485 ± 15 nm, respectively. The quality and size of electrospun fibers were highly dependent on different parameters such as viscosity, polymer concentration, solution conductivity, nanosheets particles, and type of polymer [50,51]. Increasing the solution conductivity results in the generation of much more homogeneous fibers and reduces the fibers’ diameter [48]. The polymer solution conductivity is highly enhanced by adding rGO with high electrical conductivity, increasing the electric charges, and reducing the polymer fibers diameter [52]. However, to prevent inflammatory cells from moving to the lumen and lessen the dissemination of the growth factors out of the guide lumen, the scaffolds’ pore size cannot be more than a specific size [29,51]. A corresponding histogram analysis exhibited the extended range of nanofibers diameters based on collagen fibrils (10–300 nm) in native tissue, implying the fabricated nanofibers may assist the cell growth. To disseminate oxygen, nutrients, and neurotrophic factors and prohibit the penetration of fibrous tissues, the perfect scaffolds for nerve tissue engineering should have nano to micrometer pore size in the range of 50 nm to 5 μm [13].

**Figure 4:**
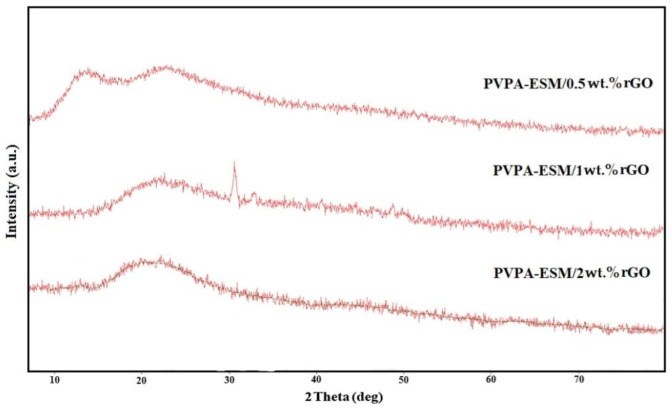
X–ray diffraction patterns of the electrospun PVPA–ESM/xwt.%rGO nanofibers (x = 0.5, 1 and 2 wt.%).

Based on the XRD patterns of the PVPA–ESM/rGO nanofibers, only two characteristic peaks were observed in the range of 11° to 25° after the crosslinking process (Figure 4). The first peak at 11.9° was attributed to the homogenous distribution of PVPA and ESM within the fibers. The second (23.4°) was related to graphene nanosheet characteristic peak [15]. According to the XRD patterns of pure PVPA and ESM, both were semicrystalline with a broad peak at 19.6° and 28.6°, respectively [26]. Both peaks vanished after mixing, and just one peek at 19.6° could be identified, which could be due to their homogeneous blending and the interactions.

### 3.3. FTIR Analysis

**Figure 5:**
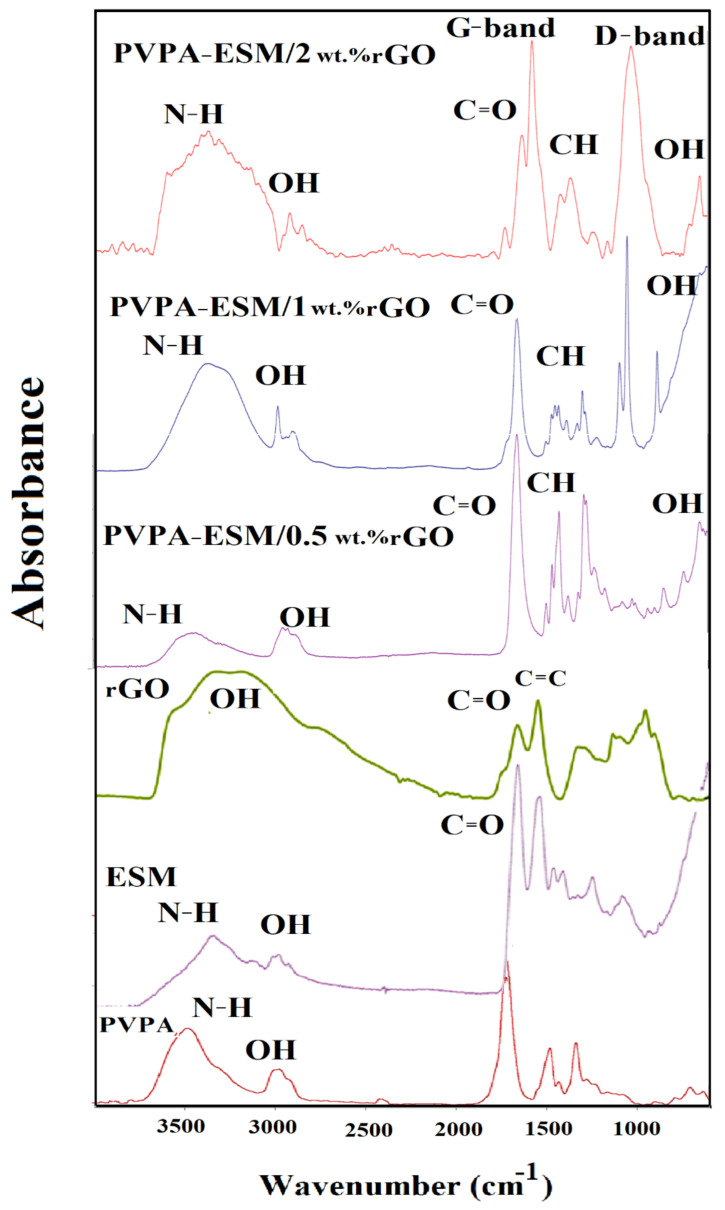
FTIR spectrum of the, PVPA, ESM, rGO, and PVPA–ESM/x wt.% rGO nanofibers with various rGO concentrations (x = 0, 0.5, 1 and 2 wt.%).

The FTIR spectrum of the PVPA, ESM, rGO, and PVPA–ESM/xwt.%rGO nanofibers were demonstrated in Figure 5. FTIR spectrum of PVPA showed the characteristic peak at 2891 cm^−1^ for CH stretching [18]. The adsorption peaks at 1312 cm^−1^ and 1631 cm^−1^ show stretching vibrations in amide band III (C–N) and carbonyl (C=O), respectively. The characteristic peaks at 1290 cm^−1^, 1660 cm^−1^, and 2921 cm^−1^ correspond to stretching vibrations in C–N, C=O, and C–H, respectively [19,20]. The FTIR spectrum of rGO exhibits characteristic peaks corresponding to the O–H vibrations at 3412 cm^−1^. Furthermore, the characteristic stretching vibrations peaks of C=O and C=C were observed at 1726 cm^−1^ and 1620 cm^−1^. A more prominent peak at 1067 cm^−1^ correspond to the C–O peak [45]. The FTIR spectrum of samples with various rGO concentrations confirmed that the rGO was incorporated into nanofibers (Figure 5). The characteristic peaks of PVPA–ESM polymer and rGO were observed in this Figure 5, which established the formation of bands between PVPA–ESM polymer composite and rGO nanosheet. The nanofibers indicated the characteristic peaks of PVPA at 1360 cm^−1^ and 1443 cm^−1^ related to O–H and C–H bonding. Furthermore, the PVPA polymer’s distinct peaks showed the crystallinity of this polymer at 1143 cm^−1^ [6]. The other peaks in Figure 5 were related to ESM polymer. The FTIR spectrum of ESM included a lot of peaks at 3278 cm^−1^, which could be ascribed to the vibration of O–H and N–H groups. The peaks at 1631, 1529 and 1236 cm^−1^ were related to a carbonyl group (C=O), amide II (C–N and N–H), and amide III of the glycoprotein mantle of the ESM, respectively [26]. The characteristic peaks of rGO nanosheet were observed at around 1580 cm^−1^ (G–band) and 1340 cm^−1^ (D–band). However, the structural defects (disorder–induced modes) of the graphene samples caused that the D– and rGO–bands to be attributed to the graphitic carbons with sp2 hybridization [53]. The rGO’s characteristic peaks were significantly decreased when dispersed in the PVPA–ESM matrix [6].

### 3.4. Mechanical Properties

**Figure 6:**
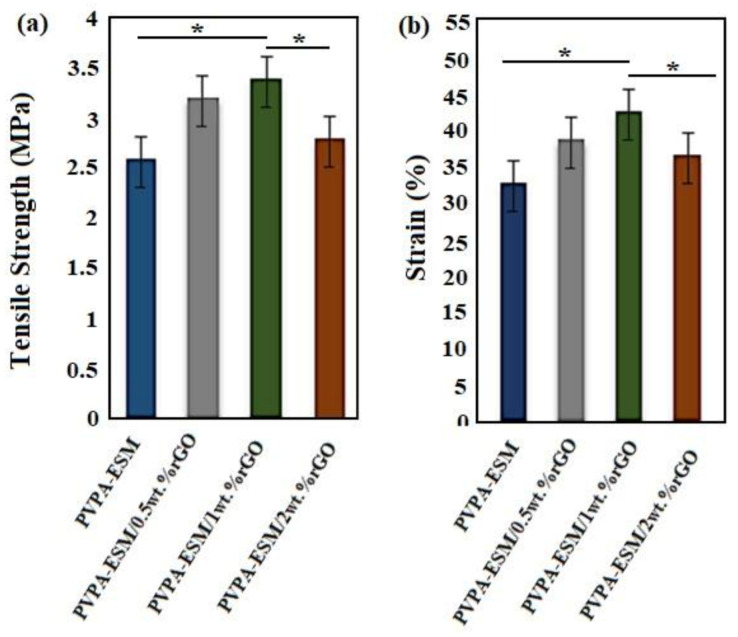
(**a**) Tensile strength, and (**b**) strain at break of PVPA‒–ESM/x wt.% rGO nanofibers with various rGO concentrations (x = 0, 0.5, 1 and 2 wt.%) (*p* * < 0.05).

The mechanical properties of wound dressings are one of the important factors due to their effects on the healing process. The evaluation of tensile strength and strain at break point is very important for initial and irradiated wound film of single–phase and composite components [54]. The tensile strength and strain of modified and unmodified samples are shown in Figure 6a,b. The composite nanofibers withstand high loads and sustain deformation when they are utilized to help the performance of tissue engineering scaffolds. The tensile strength of the nanofibers was increased by incorporation the different percentages of rGO up to 1%. Furthermore, the same behavior was observed in the tensile strain at the failure point. The tensile strength and strain of PVPA–ESM/0.5 wt.% rGO and PVPA–ESM/1 wt.% rGO were increased from 3.16 ± 0.81 MPa to 3.4 ± 0.95 MPa and 37.1 ± 3.2 to 43.2 ± 4.12%, respectively. By incorporating 2 wt.% rGO, tensile strength and strain reduced to 2.65 ± 0.71 MPa and 30.2 ± 3.51 wt.%, respectively. The tensile strength and deformation resistance of the PVPA–ESM nanofibers without rGO was 28% and 23% less than that of the sample with 0.5% rGO. Moreover, aggregation of rGO nanosheets in the PVPA–ESM/rGO composite structure occurred in the specimens with a high concentration of rGO (2 wt.%). This is due to the rougher structure and the significant difference in the diameter of fibers in samples with a higher rGO concentration. Furthermore, the high degree of agglomeration of rGO in polymeric matrix and consequently the reduction of the interaction between rGO and PVPA–ESM is another reason for the reduction of mechanical properties. Gholafshan et al. [54] synthesized a nanohybrid interpenetrating network hydrogel composed of laponite:polyvinyl alcohol (PVA)–alginate by gel casting method for wound healing application. The tensile strength, elastic modulus, elongation, and toughness of optimized laponite:polyvinyl alcohol (PVA)–alginate with 0.5 wt.% laponite was found to be 0.45 MPa, 1 MPa, 140%, and 0.35, respectively, which are still far away from the mechanical properties of PVPA–ESM–rGO composite that we fabricated in this study.

### 3.5. Contact Angle, Degradation Behavior and Swelling Results

**Figure 7:**
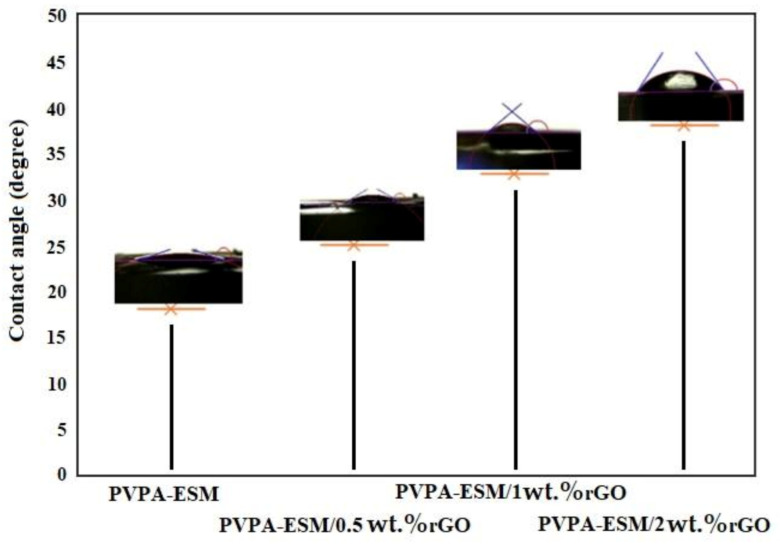
Contact angle measurements of the electro–spun PVPA–ESM/x wt.% rGO nanofibers with various rGO concentrations (x = 0, 0.5, 1 and 2 wt.%).

The water contact angle of electrospun PVPA–ESM composite nanofibers was compared with modified samples with rGO (Figure 7). A spherical water droplet with a contact angle of 17.8° was observed on the PVPA–ESM nanofibers surface. Increasing the amount of rGO to 0.5, 1 and 2 wt.% increased the water droplet’s contact angle to 25.7°, 33.8° and 38.4°, respectively. When rGO was added, the nanofibers composite showed a hydrophobic nature. It was shown that pristine rGO has a contact angle of 58° due to the oxygen–containing functional groups on their surface [53]. The PVPA–ESM–containing rGO showed an increased water contact angle. It was also reported in the literature that rGO nanosheets have had a useful role in the water impermeability of the nanofiber scaffolds [55]. These nanofibers will have a higher potential to be used in biomedical applications due to their excellent biocompatibility.

**Figure 8:**
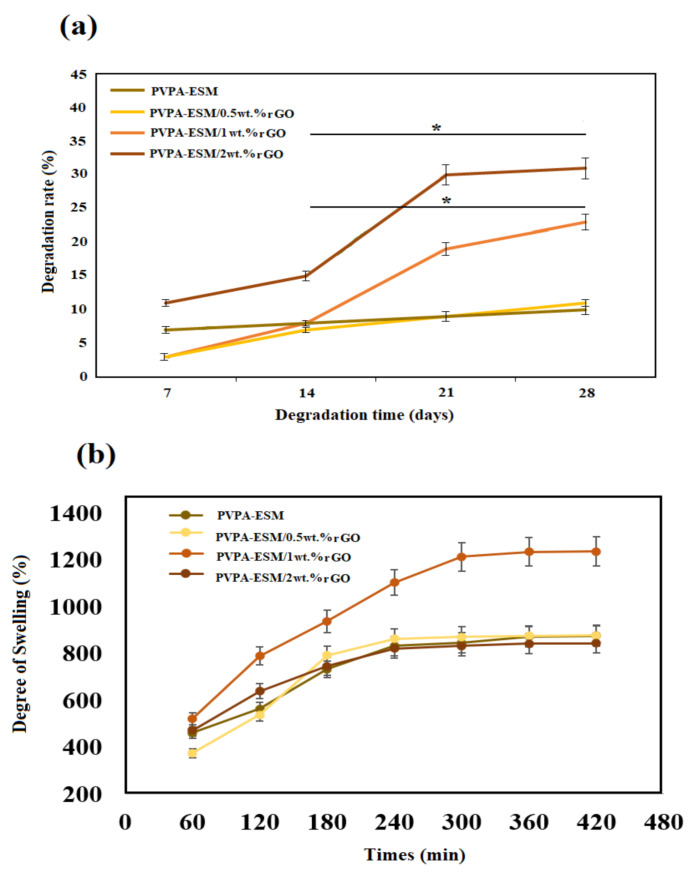
(**a**) Degradation rate and (**b**) degree of swelling of the electrospun PVPA–ESM and PVPA–ESM/x wt.% rGO nanofibers with various amount of rGO nanosheets (x = 0, 0.5, 1 and 2 wt.%) as a function of the soaking time (p * < 0.05).

Mechanical properties of implants are influenced by their degradation in the body environment, which is one of the most crucial features of hydrophilic–based nanofibers. As seen in Figure 8, there is a difference between the degradation of PVPA–ESM/rGO nanofibers with various amounts of rGO and PVPA–ESM. After 28 days of soaking in PBS, the degradation rate was reduced from 30.8 ± 2.6% (for PVPA–ESM–2 wt.% rGO) to 10.8 ± 1.1% (for PVPA–ESM/0.5 wt.% rGO) and 10.1 ± 1.1% (for PVPA–ESM). This could be due to the strong interaction between rGO and PVPA–ESM networks that can bridge the cleaved PVPA–ESM chains, causing a delay in nanofiber degradation. The composite of methacrylate rGO–gelatin methacryloyl (GelMA) hybrid hydrogel has shown the same behavior [56]. However, the degradation rate (*p* < 0.05) was significantly increased by increasing the rGO amount in the PVPA–ESM nanofibers. This is due to the interaction between rGO nanosheets and the PVPA–ESM polymer network. rGO agglomeration in samples enhanced the polymer degradation and the release of rGO. An ideal wound dressing for skin regeneration should absorb wound fluid to prevent infection of wound. A highly porous three–dimensional network of this composite has a positive effect on cell penetration and diffusion of nutrients, metabolites and other signaling molecules as well as allowing good vascularization due to its water uptake ability [57]. The degree of swelling of different wound dressing composite PVPA–ESM in various amount of rGO nanosheets was demonstrated in Figure 8b. As can be seen, the swelling rates of composites hydrogels were very fast at the initial stage. As shown in Figure 8b the sample with 0, 0.5 and 2 wt.% rGO showed the same swelling behavior and showed the lowest degree of swelling. Although the PVP is hydrophilic but does not have good absorption capacity, but when blended with rGO nano sheets, its swelling property increased significantly. It can be easily observed (Figure 8b) that all the composites went to an equilibrium condition after 300 min immersion in PBS. The maximum degree of swelling was observed for samples with 1% rGO nano sheets 1235% after 420 min. This high swelling property of PVPA–ESM/1 wt.% GO composite dressing can be explain by the presence of hydrophilic groups on the surface of rGO nanosheets. These functional groups fastened the water diffusion in the polymer networks. In contrast, increasing the rGO nanosheets to 2 wt.% reduced the swelling degree due to agglomeration of rGO nanosheets which decreased the interactions between rGO nanosheets and the polymer network. In a work done by Golaphshan et al. [54], they characterized the swelling ratio of polyvinyl alcohole–alginate–laponite. They reported that, after 24 h soaking, the swelling ratio of polyvinyl alcohole–alginate was measured as 274.6 ± 6.5%, while incorporating various amounts of laponite in this composite resulted in a decrease in the swelling ratio up to 3.2 times for composite with 2 wt.% laponite.

### 3.6. In Vitro Study

**Figure 9:**
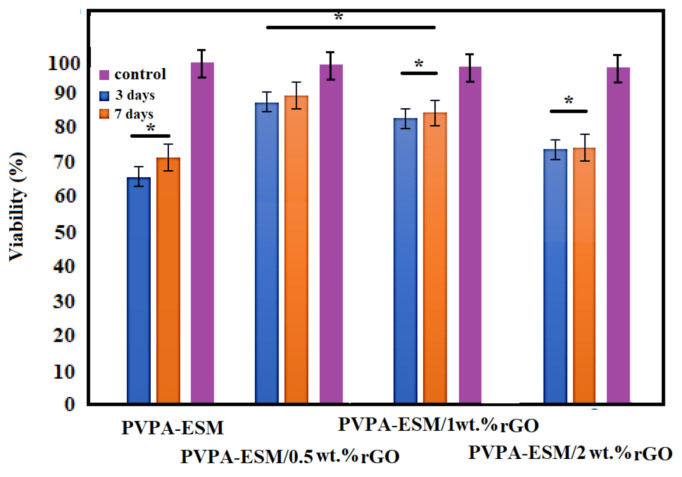
Cell viability of PVPA–ESM/x wt.% rGO nanofibers with various rGO concentrations (x = 0, 0.5, 1 and 2 wt.%) (* not significantly difference between various samples, *p* > 0.05).

The hydrogel of biocompatible wound dressings should create a suitable microenvironment and could increase cell migration, hemostatic, and adhesion, and aid angiogenesis and connective tissue regeneration. Thus, another critical factor in selecting the right biomaterials for the fabrication of wound dressings is biocompatibility [23,58]. The cytotoxicity of PVPA–ESM nanofibers with various percentages of rGO (0, 0.5, 1 and 2 wt.%) was performed using the MTT test. The cell viability of the control sample was 100%. According to the results, the cell viability of PVPA–ESM nanofibers after 3 and 7 days was 65% and 71% [58], which indicated the toxicity of PVPA–ESM (Figure 9). The cell viability percentage of PC12 cultured of modified samples after 3 and 7 days are presented in Figure 9, too. In the PV–ESM 0.5 wt.% rGO and PV–ESM 1 wt.%rGO samples, the rate of cell viability was above 85% (after both 3 and 7 days), which indicated the nontoxic behavior of those samples. For the sample with 2 wt.% rGO, the cell viability percentage after 3 and 7 days was 73 and 74%. A considerable reduction (*p* < 0.05) of cell viability was observed in the PVPA–ESM/2 wt.% rGO nanofibers based on the statistical analysis results. Based on the MTT assay, the toxicity of the PVPA–ESM sample was significantly reduced by the incorporation of 0.5 wt.% rGO compared to other groups. As observed, various factors, including the type of nanosheets particles, pH changes in the culture medium, and the time of culture, affected the cell viability. The presence of rGO in the modified samples and the release of rGO in the medium were the main reason of increased cell viability. However, note that by increasing the release of rGO nanosheets in the culture medium, a reduction in cell viability was observed due to the toxicity effect of the agglomerate rGO with a high concentration and subsequent direct connection of rGO, which resulted in cell destruction. However, the culture time extended from 3 to 7 days led to the enhanced cell viability exposed to PVPA–ESM/x rGO extracts. According to Golafshan et al. [6], the cytocompatibility and proliferation of PC12 cells can be improved by including nanofillers, especially carbon nanofiber, in comparison to the PCL scaffolds. In this regard, it was also reported [59] that because of the forceful attraction between nanomaterials and polymers, cell adhesion and proliferation increased by incorporating carbon–based nanomaterials into natural substrates [60]. In another study by Jeong et al. [61], the adhesion, growth, and morphology of cultured breast cancer cells on a silicon substrate—graphene oxides (rGO)—were investigated. Based on their results, it was observed that the spreading area and number of cells were highly dependent on the hydrophobicity and the presence of oxygenated groups on rGO and Si substrates, suggesting hydrophobicity–driven cell growth. Adding the rGO nanosheets results in controlling the adhesion and growth of cells on the surface of samples [61].

## 4. Conclusions

To assist skin regeneration, graphene oxide (rGO)–loaded polyvinylpyrrolidone/eggshell membrane (PVPA–ESM) nanofibers were fabricated via electrospinning. The nanofibers had a porous and interconnected morphology featuring bead–free and randomly aligned continuous nanofibers. The rGO content increase enhanced the degradation behavior, mechanical strength, and deformation resistance, but mechanical features were reduced by adding 2 wt.% rGO into the PVPA–ESM nanofibers. The results confirmed that the incorporation of 2 wt.% rGO within the interpenetrating network of PVPA–ESM, respectively increased and reduced its degradation and swelling ratio. According to the MTT assay results, in the pristine PVPA–ESM and PVPA–ESM–rGO nanofibers with more rGO, cell survival was lower. These results showed that increasing the rGO content in the sample enhances the cytotoxicity.

## Data Availability

The raw/processed data required to reproduce these findings cannot be shared at this time as the data also forms part of an ongoing study. If you need to have access to some of the raw data, please contact the corresponding author.
